# A starring role for inference in the neurocognition of visual narratives

**DOI:** 10.1186/s41235-021-00270-9

**Published:** 2021-02-15

**Authors:** Neil Cohn

**Affiliations:** Department of Communication and Cognition, Tilburg School of Humanities and Digital Sciences, Tilburg University, P.O. Box 90153, 5000 LE Tilburg, The Netherlands

**Keywords:** Visual language, Inference, Visual narrative, Discourse, Comics

## Abstract

Research in verbal and visual narratives has often emphasized backward-looking inferences, where absent information is subsequently inferred. However, comics use conventions like star-shaped “action stars” where a reader *knows* events are undepicted *at that moment,* rather than omitted entirely. We contrasted the event-related brain potentials (ERPs) to visual narratives depicting an explicit event, an action star, or a “noise” panel of scrambled lines. Both action stars and noise panels evoked large N400s compared to explicit-events (300–500 ms), but action stars and noise panels then differed in their later effects (500–900 ms). Action stars elicited sustained negativities and P600s, which could indicate further interpretive processes and integration of meaning into a mental model, while noise panels evoked late frontal positivities possibly indexing that they were improbable narrative units. Nevertheless, panels following action stars and noise panels both evoked late sustained negativities, implying further inferential processing. Inference in visual narratives thus uses cascading mechanisms resembling those in language processing that differ based on the inferential techniques.

## Introduction

Inference has been a primary focus of studies of discourse (McNamara and Magliano 2009), especially in research on how we understand visual narratives, like comics or picture stories (Cohn 2020; Loschky et al. 2020; McCloud 1993; Saraceni 2016). Research on verbal narrative has often used visual narratives as stimuli, assuming them to involve or evoke similar inferential processing (Gernsbacher et al. 1990; Loschky et al. 2020; Magliano et al. 2019), and visual narratives have even been posited as tools for bootstrapping verbal inferential abilities (Kendeou et al. 2020). Yet, little research exists on the neurocognition of visual narrative inferencing itself. Thus, studying how inferences are generated in sequential images can be informative for understanding such processing across domains. This study thus asks: what are the neurocognitive correlates of inference generation, particularly when an impoverished narrative unit explicitly signals omitted information?

Much work on inferential processing in both modalities has emphasized bridging inferences, where a reader must infer the absent event information from what is explicitly provided. This is usually taken as a *backward-looking process*: a reader realizes the discontinuity between the incoming information and the prior context, and then works to “fill in” that missing information (McNamara and Magliano 2009). Such backward-looking processes have long been demonstrated in research on verbal discourse (McNamara and Magliano 2009), and comparable inferential processing has been posited across both verbal and visual narratives (Gernsbacher et al. 1990; Loschky et al. 2020; Magliano et al. 2019). Indeed, as in studies of verbal discourse (McKoon and Ratcliff 1992), costs related to inferential processing manifest at the image following omitted-information as longer viewing times and increased visual search processes (Huff et al. 2020; Hutson et al. 2018; Magliano et al. 2015, 2017). Such costs are modulated by interference to both linguistic and visuospatial working memory processes (Magliano et al. 2015), again suggesting a connection between the mechanisms involved in verbal and visual narratives.

**Fig. 1:**
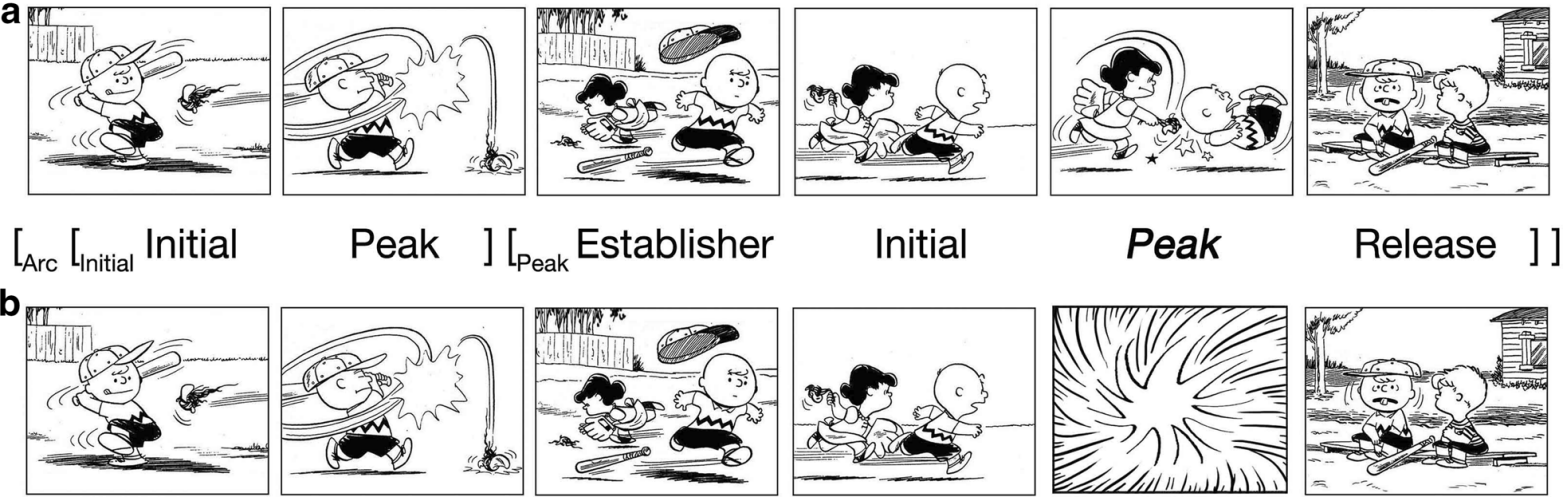
Visual narrative with either an a) explicit climactic event, or its substitution by b) an action star which omits the event but still provides a narrative climax. *Peanuts* is © Peanuts Worldwide LLC

However, consider Fig. 1b, where the penultimate panel uses the visual narrative convention of an “action star” (Cohn 2013, 2019), whereby the climactic event (Lucy hitting Charlie with a beat-up baseball) remains unseen (as in Fig. 1a). With an action star, a reader *knows* an absent climactic event occurs *at that moment,* rather than events being omitted with no climatic unit at all. Action stars thus differ from situations using the canonical backward-looking bridging inferences because no image is “missing,” but rather a visual morpheme implies an event without showing it. This star shape typically depicts an impact or collision, but also can signal loudness when surrounding text like an onomatopoeia, which often co-occurs with action stars (Manfredi et al. 2017). As they are conventionalized, action stars are a panel-level unit “lexicalized” within the visual language used in comics that allow authors to leave out information and thereby sponsor reader engagement via the resulting inference (and/or providing a way for artists to avoid drawing complex climactic events). Such engagement has been posited to enhance readers’ immersion and likeability of narratives (Herman 2009; McCloud 1993; Zwaan 2004).

Yet, action stars do not just signal missing content, they also play a specific role in a sequence, since they omit climactic events specifically. *Visual Narrative Grammar* (VNG) posits that a narrative structure operates in parallel to semantics, where it organizes meaning at a discourse level (Cohn 2013, 2020). The canonical narrative schema progresses through narrative roles where the climactic moment—the Peak—contains the crucial information of the sequence. This importance is suggested because participants choose to omit Peaks at lower rates, and recognize Peaks when missing at higher rates, than other narrative categories (Cohn 2014; Magliano et al. 2017).

Action stars thus fulfil a narrative role as a Peak, letting the sequencing structure remain well-formed with a unit devoted to a climax, while still pushing the reader to generate an inference for the un-depicted events. This structural role is suggested by viewing times that are actually *faster* for action stars than blank panels in Peak position when both omit events (Cohn and Wittenberg 2015). Since action stars play a narrative role as Peaks, they facilitate processing more readily than “incongruous” panels with no content at all. Yet, consistent with other studies of visual narrative inference, panels following action stars are viewed slower than those following explicit events, implying a greater cost for construing the omitted events. Thus, both action stars and event-omission may warrant a backward-looking bridging inference at the subsequent panel, but action stars provide an explicit cue that signals to a reader that inference generation is necessary, but without omitting a narrative unit.

Nevertheless, the neurocognition of such inferencing remains understudied, though research on event-related brain potentials (ERPs) implicate similar mechanisms in visual narratives as in language processing (Cohn 2020). When encountering a panel in a visual narrative sequence, a comprehender first extracts the relevant information from a panel (Loschky et al. 2018). Specific visual cues are identified as relevant for the understanding of the sequence, given the prior context (Foulsham et al. 2016; Hutson et al. 2018; Loschky et al. 2020). These cues might be part of characters, especially faces and postures (Foulsham and Cohn 2020; Hutson et al. 2018; Laubrock et al. 2018; Loschky et al. 2020), but in action stars they are the lines comprising this abstract symbol.

Information extracted from the visual surface then activates semantic memory to process the meaning of the immediate representation in relation to what came before in the sequence. This process is indexed in ERPs by the N400, a negative deflection that peaks around 400 ms after the onset of a word or image (Kutas and Federmeier 2011; Kutas and Hillyard 1980). The N400 has been taken to reflect the brain’s default process of semantic access and/or integration, modulated by the expectancy of an incoming stimulus given the preceding context (Baggio 2018; Hagoort 2017; Kuperberg 2016; Kutas and Federmeier 2011; Nieuwland et al. 2020). As a result, larger N400 effects are shown to incongruous or unexpected images in a visual narrative sequence (or words in a sentence) than congruous ones (Coderre et al. 2020; West and Holcomb 2002). Indeed, greater N400s have been shown to bridging inferences in studies of language (Kuperberg et al. 2011; St. George et al. 1997). Though the N400 is taken to index a modality-general mechanism, visual images typically evoke another negativity peaking around 300 ms, preceding the N400, deemed the N300 (McPherson and Holcomb 1999). The N300 has been associated with object categorization or identification (Hamm et al. 2002) prior to the general semantic access indexed by the N400 (Draschkow et al. 2018; Hamm et al. 2002; Võ and Wolfe 2013).

The information activated in semantic memory then becomes incorporated into a *situation model* (McNamara and Magliano 2009; van Dijk and Kintsch 1983), a growing mental understanding of the unfurling scene. With each unit of a (visual) discourse, comprehenders may anticipate subsequent information, both for broad expectancies (ex. expecting the same characters to appear in subsequent frames) and for more specific ones (ex. predicting specific subsequent events). As the narrative progresses, discontinuities trigger an update to integrate or revise the situation model with this new information. Greater changes thus lead to increased updating across shifts between characters, spatial locations, events, and other aspects of the discourse (Zwaan and Radvansky 1998).

While situation model construction progresses in an ongoing and incremental manner across the units of (visual) narratives (Huff et al. 2014; Magliano and Zacks 2011), more significant discontinuities may warrant further processing. These cases include contexts where a reader may be motivated to fill in information that is not provided directly, thereby leading to the generation of inference. For example, in Fig. 1b, upon reaching the final panel, an inference would be expected to resolve the incongruity of the missing climactic event. Thus, bridging inferences can be viewed as a process of situation model construction triggered in the absence of information provided overtly.

In ERPs, updating or revision processes have been indexed by the P600, a positive deflection posteriorly distributed across the scalp that typically onsets after the time window of the N400 (Brouwer et al.; Kuperberg 2016; Van Petten and Luka 2012). Though originally evoked by syntactic violations or ambiguities in language (Hagoort et al. 1993; Osterhout and Holcomb 1992), P600s have now been associated with processes related to the revision or integration of a structure given the relation between incoming information and its previous context (Baggio 2018; Brouwer et al. 2016; Kuperberg 2016), possibly tied to more general cognitive updating processes (Donchin and Coles 1988; Leckey and Federmeier 2019). In visual narratives, P600s have been observed to both incongruous and congruous changes between panels to events, characters, or the framing of information (Cohn 2020; Cohn and Foulsham 2020).

Nevertheless, P600s may not index inference processes specifically. Only one ERP study has investigated inference processing in visual narratives (Cohn and Kutas 2015), which compared sequences showing explicit events with those showing a character watching an event off-panel (such as Charlie Brown watching Snoopy chase after a ball he threw). A final panel then confounded the expectations of the narrative sequence (like Linus returning with the ball in his mouth instead of Snoopy). Here, the “onlooker” (Cohn 2019) of the off-panel event either cued a climactic Peak (e.g., with a surprised expression) or lacked such cues (e.g., with a neutral facial expression). The cued-Peaks evoked a larger P600 than those without such a cue, taken to reflect greater updating of a situation model given the explicitness of the cued-observer’s actions, and indeed even larger P600s appeared to explicit events. Thus, while the P600 may reflect an updating of the situation model, it cannot solely indicate inferential processes at this position, as it appears even greater to non-inference demanding representations, all of which were congruous in the sequence context.

Indeed, at the subsequent panel which clarified the off-panel event, a P600 was suggested only following the non-cued onlooker, despite both cued and non-cued versions eliciting the same inference. The P600 in this context was taken as an indicator of the difference in narrative structure, since the inferential content was held constant, with the preceding cued panel being more suggestive of a climactic Peak than the non-cued panel. Indeed, P600s have appeared to disruptions of the narrative grammar alone, with no manipulations of meaning (Cohn et al. 2014) suggesting a process of structural reanalysis or revision in line with P600s originally shown to violated syntactic structure sentence processing (Hagoort et al. 1993; Osterhout and Holcomb 1992). Such findings further support that the P600 may operate across both grammatical and semantic processing, or at their interface (Bornkessel-Schlesewsky and Schlesewsky 2008; Brouwer et al. 2016; Michalon and Baggio 2019).

Rather, inferential processes in this study may have instead been indexed by sustained anterior negativities which were evoked by panels following both cued and non-cued onlookers (Cohn and Kutas 2015). Such sustained negativities often with a more central or frontal distribution, have been posited in studies of verbal discourse to reflect interpretive processes subsequent to the relational semantics indexed by the N400 (Baggio 2018; Baggio et al. 2008; Bott 2010; Hoeks and Brouwer 2014). Such negativities may index working memory processes seeking to resolve complex ambiguities, like inferences (van Berkum 2009), or a mechanism searching through a mental model (Hoeks and Brouwer 2014). Sustained negativities have been observed to visual narratives following the N400 time window in most all studies of semantic processing looking at sequential images (Cohn et al. 2012; West and Holcomb 2002).

**Fig. 2:**
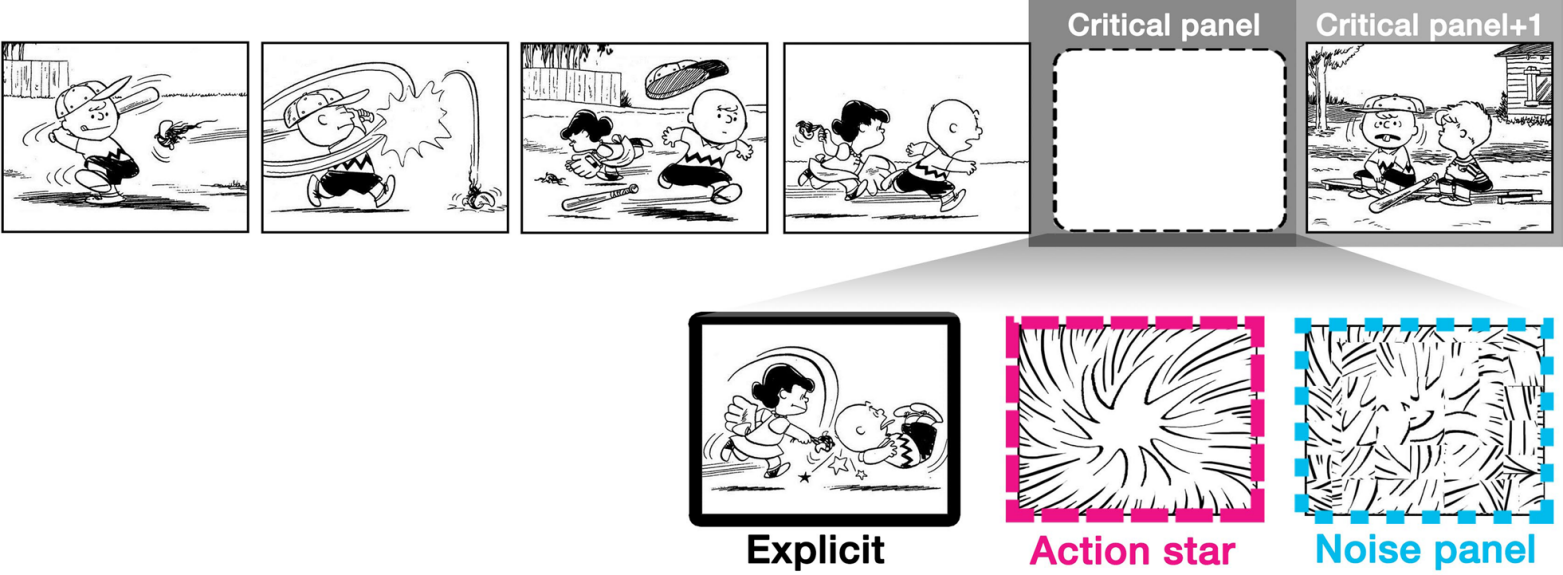
Example stimuli for visual narrative sequences with a critical panel of either an explicit event, a conventionalized action star, or a noise panel. *Peanuts* is © Peanuts Worldwide LLC

In comparison with onlookers, action stars show no explicit referential information at all—they depict only a symbolic star-shape—while directly cuing both narrative and inference (Cohn 2019). Thus, here we compared sequences depicting explicit events with those substituted with action stars. These conventional sequences were further contrasted with sequences substituting climactic events with “noise panels”—panels created by scrambling the lines from action stars into non-representational configurations (Fig. 2).

Given prior theories of visual narrative processing (Cohn 2020; Loschky et al. 2020), the action star should demand relatively few costs of information extraction, because of its simple symbolic representation, compared to more complex depictions of events. Such information extraction would thus be impossible for noise panels, which depict no meaningful cues at all. From here, semantic access (N400) should become harder for action stars and noise panels, because the surface information will have less feature overlap with explicitly depicted events. If the event-cues of action stars sponsor an inference more than the noise panels, and if such an inference is manifest in the access to semantic memory, we might expect an attenuated N400 to action stars. However, both the action star and noise panels could equally sponsor inference at this position, as in findings that N400s did not differ to onomatopoetic (*Pow!*) and descriptive words (*Punch!*) in action stars, which were both attenuated compared to anomalous words, suggesting that they were both sufficient as event-cues given the context of the prior visual sequence (Manfredi et al. 2017).

Regardless of their relational semantics, the event-cues of an action star should better contribute to constructing a situation model than a noise panel, which cues no semantic information at all. Thus, a greater P600 should appear to action stars than the noise panel, indexing the cost of updating or revising a situation model. Such a finding would be consistent with prior work showing a larger P600 to onlookers with cues suggestive of off-panel events compared to those without such cues (Cohn and Kutas 2015). This may provide evidence that action stars trigger an update of information into a situation model, even if content is not provided outright.

We might also predict updating processes at the panel following an action star or noise panel, as further explicit information should demand the inference of the missing content. This has been suggested behaviorally by longer viewing times following action stars and the omission of climactic events (Cohn and Wittenberg 2015; Hutson et al. 2018; Magliano et al. 2015). Nevertheless, prior work has not shown a P600 to inference-following panels in visual narratives, where a P600 appeared only to a *narrative* difference between panels where the inference was held constant. Rather, a frontally distributed negativity appeared to both types of inference generating panels, taken as possibly indicative of working memory processes, in line with sustained negativities in language to sentence- and discourse-level inferences (Baggio 2018; Baggio et al. 2008; Bott 2010; Hoeks and Brouwer 2014). Thus, sustained negativities at the panel following action stars or noise panels would also be consistent with inferential processing.

## Materials and methods

### Stimuli

We used visual sequences constructed out of panels from *The Complete Peanuts* which have been used in prior studies of visual narrative comprehension (e.g., Cohn et al. 2012; Cohn and Wittenberg 2015). All sequences were 6-panels long, had no text, and were normed to a consistent size. To ensure that these sequences had strong potential for inference of a missing Peak panel, our stimulus selection was guided from data from a norming study. There, 101 participants (54 female; mean age: 40.8, range: 23–65; mean VLFI: 20.8, range 2.5–49) viewed 120 sequences where one panel had been omitted, and they were asked to identify where a panel had been missing. This allowed us to assess the rate by which participants may be accurately inferring missing content, relevant here for Peak panels specifically. We therefore chose 60 sequences from this prior study to use in this experiment. Our final sequences had a mean of 0.71 (SD = 0.16) accuracy for participants recognizing that the Peak panel was omitted.

From these strips, we created three sequence types, as in Fig. 2. Our event-*explicit* sequences used the original sequence with Peak panels intact. Critical panels fell in various ordinal positions so that participants could not predict its location, including the second panel in the sequence (2 strips), third panel (5), fourth panel (13), and fifth panel (40). Action star sequences substituted an action star panel for the explicit Peak panels. Because action star panels can have less visual information than explicit panels, we therefore designed action star panels with a similar degree of visual complexity as our explicit Peak panels. We calculated the “gray value” of all pixels across all explicit Peak panels (*M* = 213.2) and designed three action star panels with similar values (*M* = 213.0). These three action star panels were distributed randomly across sequences within the action star sequence types. Our final sequence type then created “noise” panels by selecting subdivisions of action star panels (via a grid and iterated circular pixel selections), and then rotating these subdivisions randomly such that no discernable representational information was conveyed, though the quantity and character of the visual lines remained the same. Action stars were used as the base for noise panels rather than explicit panels in order to ensure they remained asemantic, and also involved similar line segments. Noise panels had a grey value comparable to other critical panels (*M* = 213.0).

These 60 sequences were counterbalanced into three lists using a Latin Square Design such that participants viewed each sequence only once, but across lists all participants viewed all sequences in all conditions. An additional 96 fillers, half of which contained sequencing discontinuities, were used to introduce additional heterogeneity into the experimental stimuli.

### Participants

We recruited 24 participants from Tilburg University (14 male, 10 female; mean age: 23.8, SD = 4.9). Although statistical power was not computed a priori, a power analysis in G*Power indicated that with 3 conditions in a sample of 24 participants, to achieve a medium effect size of 0.25, it would require *F*-values above 3.2 for our within-subjects design. All participants gave their informed written consent prior to the experiment and the study was approved by the Tilburg University School of Humanities and Digital Sciences Research Ethics and Data Management Committee. Participants were right-handed, taking no psychiatric medications, with no history of head trauma, and with normal or corrected-to-normal vision. Prior to experimentation, participants filled out the Visual Language Fluency Index (VLFI) questionnaire which assessed their frequency of reading and drawing visual narratives like comics, along with experiencing other media like movies and written books. The VLFI score generated by this assessment has been a consistent predictor of individual differences in visual narrative comprehension (Cohn and Kutas 2015; Cohn and Maher 2015; Cohn et al. 2012; Cohn and Wittenberg 2015). Participants in this study overall had a mean VLFI score of 14.14 (SD = 5.7, range 3–24.7), which is considered average (high: > 20, average: ~ 12, low: < 8) and consistent with average scores in prior ERP studies.

### Procedure

The experiment was conducted in a soundproof chamber, where a participant sat in a chair across from a computer screen. Lights in the chamber were turned off, except for backlighting behind the screen used to prevent a flashing effect of stimuli on the screen that could induce blinks. We used PsychoPy2 (Peirce et al. 2019) to present experimental trials, which began with a screen reading “Ready” in white letters on a grey background, where participants pressed a button to start each trial. A red dot persisted in the center of the screen to provide participants with a sustained fixation point. Once participants pressed to begin a trial, each black-and-white panel appeared one at a time in the center of the otherwise grey screen at a size of 10.16 × 8.04 cm, yielding a visual angle of 5.2° horizontally and 4.2° vertically. Each panel remained on screen for a duration of 1350 ms, separated by a 300 ms ISI to prevent a “flip book” effect of panels appearing to be animated. These durations are consistent with prior ERP studies of visual narratives (Cohn et al. 2014; Cohn and Kutas 2015, 2017; Cohn and Maher 2015). At the end of each sequence, a question mark cued participants to rate the comprehensibility of the sequence on a 1 (= hard to understand) to 7 (= easy to understand) Likert scale.

Following all experimental trials, participants filled out a post-test questionnaire which probed their observations of stimuli, which they could fill in with open-response answers. This questionnaire asked: “Did you notice any patterns in the comic strips that you saw? Did you notice anything unusual about any of the comic strips? If yes, what do you think made them unusual? Were there aspects of these unusual strips that were different from each other? Why do you think they were different (or similar)?”.

### Data analysis

**Fig. 3:**
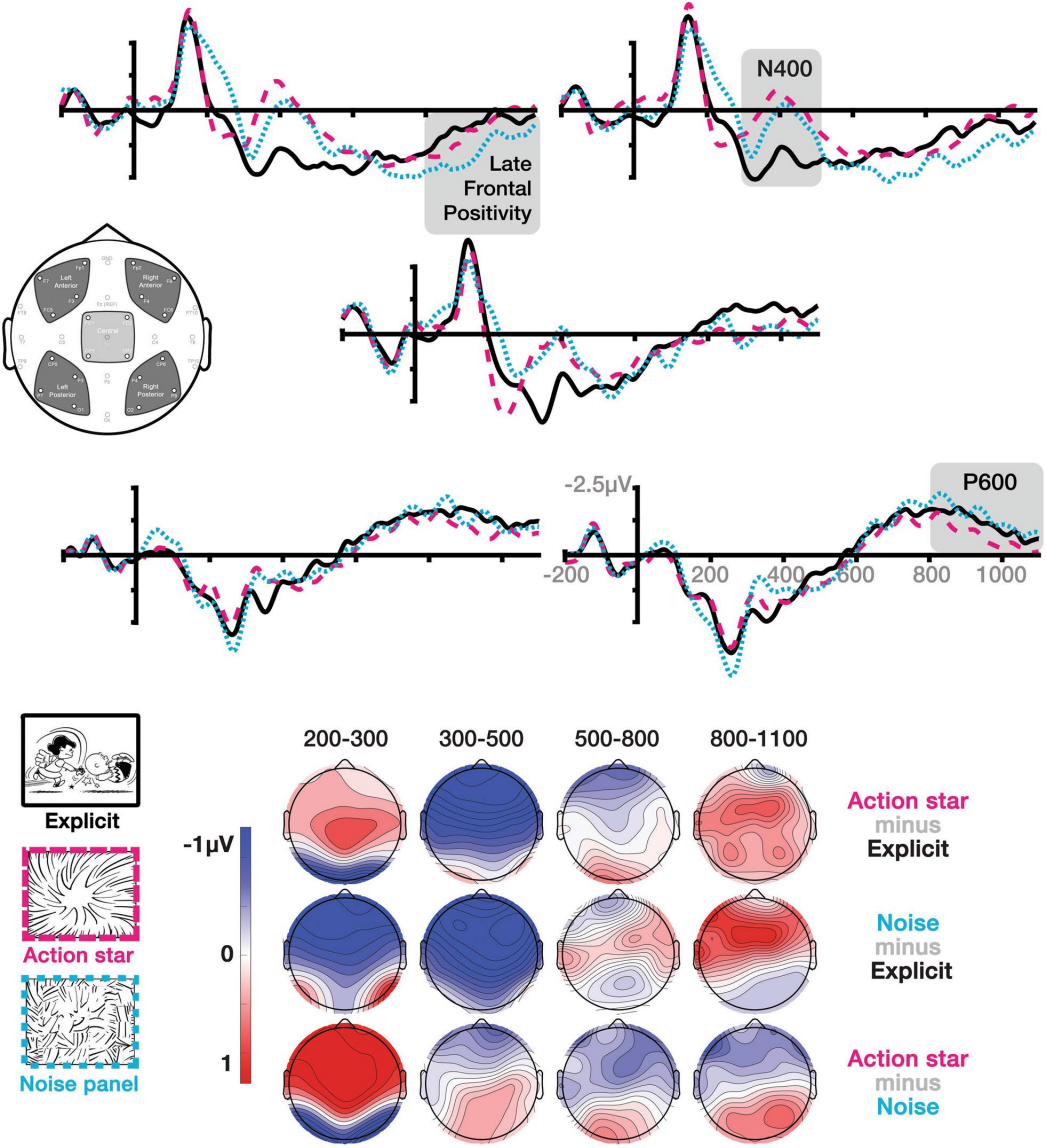
ERPs time-locked to the critical panel of explicit event panels, action stars, and noise panels represented across electrodes and topographic maps. Highlights show analyzed epochs of the primary effects of the N400, P600, and Late Frontal Positivity. Negative is plotted upwards

**Fig. 4:**
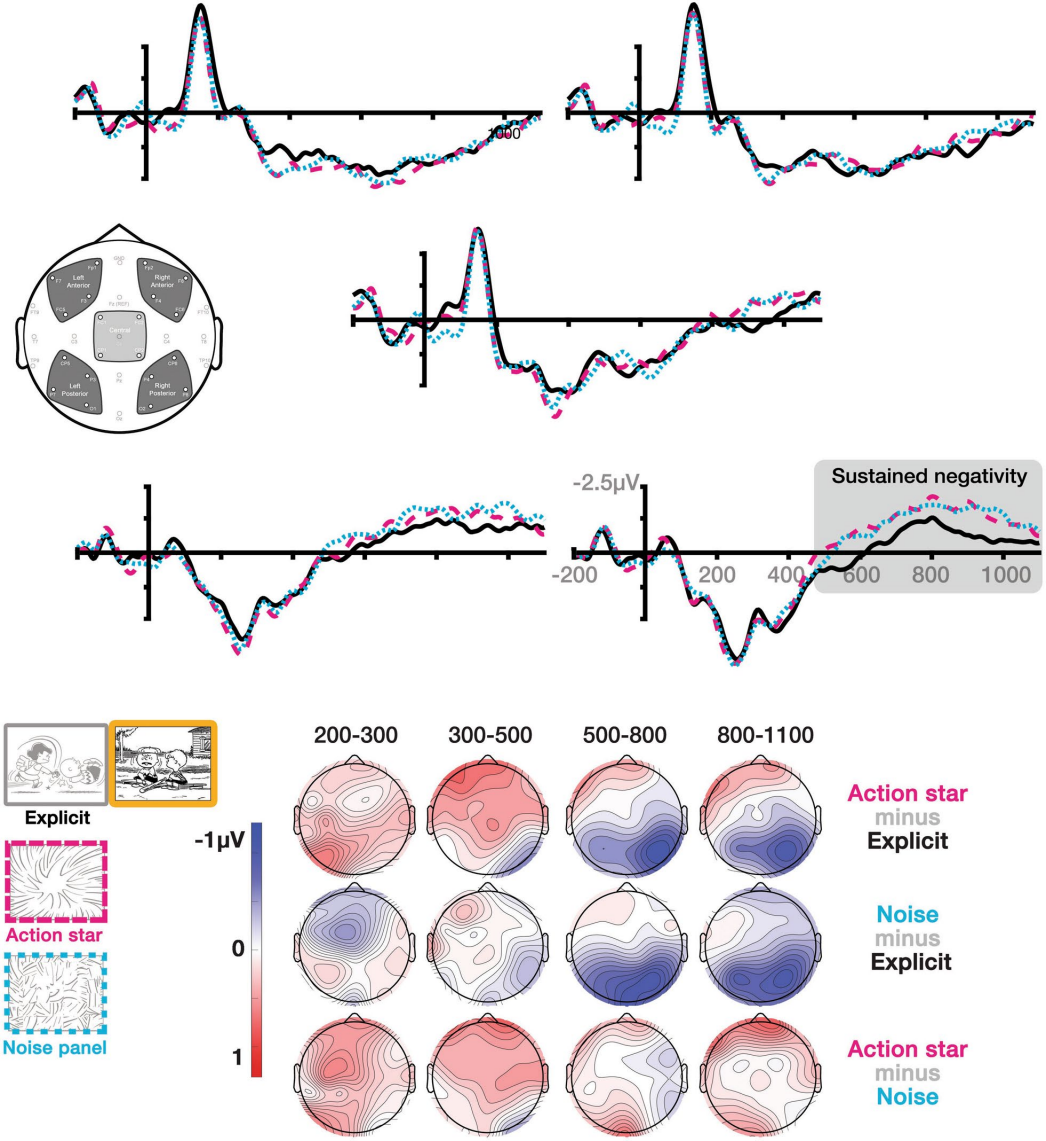
ERPs time-locked to the critical panel + 1, i.e., the panel following explicit event panels, action stars, and noise panels represented across individual electrodes and topographic maps. Highlight shows the analyzed epoch of the sustained negativity. Positivity Negative is plotted upwards

EEG was recorded using a Brain Products ActiChamp system at a sampling rate of 250 Hz and high cutoff filter of 70 Hz. EEG recordings were made with 32 channel Standard actiCAPs, referenced online to electrode Fz. Eye movements and blinks were monitored using electrodes placed beneath the right eye and beside the left eye. Electrode impedances for all electrodes were kept below 10 kΩ. We analyzed the data using the ERPLAB plugin for EEGLAB in MATLAB (Lopez-Calderon and Luck 2014). EEG data was refiltered offline with a bandpass filter of 0.1–30 Hz, and re-referenced offline to the average of the mastoid channels (TP9, TP10). An additional filter of 0.1–15 Hz was applied for presentation of data in Figs. 3 and 4, but this filtered data was not analyzed.

Trials with excessive blink or muscle artifact were isolated and removed. Across conditions at the critical panels, 6% (range 3–8%) of trials were rejected per participant, an average of 1.25 trials (18.75 trials retained). A 3 (explicit, action star, noise panel) × 2 (critical panel, critical panel + 1) ANOVA showed no significant differences in the rejection rates across sequence types (*p* = 0.246), critical panel position (*p* = 0.095), or their interaction (*p* = 0.210).

Our analysis focused on the ERPs to the critical, manipulated panel, and to the subsequent, critical panel + 1. ERP amplitudes across sequence types were compared in the epochs of 200–300 ms, 300–500 ms, 500–800 ms, and 800–1100 ms corresponding to the ERP components of the N300, N400, and later effects (sustained negativities, P600, late frontal positivity). Additional analyses in epochs of 0–100 ms and 100–200 ms investigated the potential for early, stimulus-driven physical differences at the critical panels. A broad coverage of the scalp was examined using five regions of interest which each averaged the amplitudes across four electrodes. As in Figs. 3 and 4, these included a central region (FC1, FC2, CP1, CP2), and peripheral regions of the left anterior (Fp1, F7, F3, FC5), right anterior (Fp2, F8, F4, FC6), left posterior (CP5, P3, P7, O1), and right anterior (CP6, P4, P8, O2). Statistical analyses for each critical panel and each epoch used repeated-measures ANOVAs with factors of Sequence Type (3 levels: explicit, action star, noise panel), and additional factors in the peripheral regions of Hemisphere (2 levels: left, right) and Anterior–Posterior (AP) Distribution (2 levels: anterior, posterior). Significant interactions in the omnibus analyses of peripheral regions were followed by repeated measures ANOVAs comparing Sequence Types in each region with post-hoc pairwise contrasts using a Bonferroni correction.

Behavioral results were compared using a repeated-measures ANOVA with three levels of Sequence Type (explicit, action star, noise), with post hoc tests using a Bonferroni correction. Finally, to investigate any possible influence of comic reading experience, we used Pearson's correlations with an alpha level set to 0.05 between VLFI scores and ratings and the mean amplitude differences averaged across all electrode sites at the critical and subsequent panels.

## Results

### Behavioral results

Analysis of participants’ ratings suggested differences in the sequences’ comprehensibility, *F*(2,46) = 12.54, *p* < 0.001. Explicit sequences (*M* = 5.3, SD = 1.1) were rated as more comprehensible than sequences with action stars (*M* = 4.5, SD = 0.94) or noise panels (*M* = 4.6, SD = 0.87), all *t*s > 4, all *p*s < 0.005, but these latter types did not differ from each other (*p* = 1). Nevertheless, all sequences were rated as comprehensible on the whole, rated above the midway point of 4 on a 1–7 scale (all *t*s > 2.6, all *p*s < 0.05). Finally, in open response post-test questionnaires, 35% of participants commented on observing action stars, while 33% noticed noise panels and that they differed from action stars (ex. “some pictures were more like an explosion, others looked like wires”), including one who drew a noise panel.

### Critical panel

We first analyzed ERPs at the critical panels, depicted in Fig. 3. All results from omnibus analyses are provided in Table 1. In the 0–100 ms epoch, an interaction arose between Sequence Type and Hemisphere. However follow up analyses with ANOVAs in each region resulted in main effects of Sequence Type which did not exceed the threshold of significance (all *F*s < 2.7, all *p*s > 0.079, all *η*^2^_p_ < 0.105). No significant differences arose in the 100–200 ms epoch.

**Table 1:** Results of ANOVAs comparing sequence types at the critical panel and critical panel + 1 for central and peripheral regions of interest

	Sequence type (ST)				ST*H		ST*AP		ST*H*AP	
	Central				Peripheral					
	*F*	*η*^2^_*p*_	*F*	*η*^2^_*p*_	*F*	*η*^2^_*p*_	*F*	*η*^2^_*p*_	*F*	*η*^2^_*p*_
*Critical panel*										
0–100	1.9	0.08	1.9	0.08	4.4*	0.16	0.3	0.013	0.7	0.03
100–200	0.48	0.02	0.04	0.002	0.03	0.001	0.99	0.04	0.39	0.02
200–300	6.8**	0.23	1.7	0.07	18.3***	0.44	0.54	0.02	0.02	< .001
300–500	7.3**	0.24	7.6***	0.25	8.8***	0.28	0.4	0.02	0.15	0.01
500–800	1.1	0.05	1.6	0.06	7.4**	0.24	2.1	0.08	3.5*	0.13
800–1100	1.5	0.06	1.3	0.05	8.1***	0.26	2.4	0.09	0.15	0.01
*Critical panel* + *1*										
200–300	0.21	0.01	0.26	0.01	0.75	0.03	0.27	0.01	0.33	0.01
300–500	0.43	0.02	0.21	0.009	3.6*	0.13	0.91	0.04	0.59	0.03
500–800	0.42	0.02	0.89	0.04	6.5**	0.22	4.2*	0.15	0.02	< .001
800–1100	1.7	0.07	1.9	0.08	6.6**	0.22	2.7^	0.1	0.77	0.03

*ST* sequence type, *H* hemisphere, *AP* anterior–posterior distribution

^ *p* < .1; * *p* < .05; ** *p* < .01; *** *p* < .001. *df* = 2,46

In the 200–300 ms epoch, a main effect of Sequence Type arose in the central region and interactions in the peripheral regions were found between Sequence Type and Hemisphere. Follow up ANOVAs in each region revealed main effects of Sequence Type in both anterior regions (all *F*s > 7.6, all *p*s < 0.001, all *η*^2^_*p*_ > 0.25) and in the right posterior region, *F*(2,46) = 3.3, *p* < 0.05, *η*^2^_*p*_ = 0.13. Pairwise comparisons suggested that noise panels evoked greater negativities than both explicit panels and action stars in the central and anterior regions (all *p*s < 0.05).

In the 300-500 ms epoch, main effects of Sequence Type appeared in both central and peripheral regions. An additional Sequence Type × Hemisphere interaction was followed by analyses revealing main effects in both anterior regions (all *p*s > 8.4, all *p*s < 0.001, all *η*^2^_*p*_ > 0.42). Across central and anterior regions, pairwise comparisons showed a greater negativity indicative of an N400 appeared to action stars and noise panels than explicit panels (all *p*s < 0.05), but no difference between action stars and noise panels.

Later effects were first suggested in the 500-800 ms epoch by Sequence Type × Hemisphere and Sequence Type × Hemisphere × AP Distribution interactions in the peripheral regions. Main effects of Sequence Type were subsequently found in both anterior regions (all *F*s > 6.7, all *p*s < 0.01, all *η*^2^_*p*_ > 0.149). Pairwise contrasts suggested that action stars evoked a greater negativity than both explicit and noise panels in the left anterior region (all *p*s < 0.05), and a greater negativity than the noise panels alone in the right anterior region (*p* < 0.05).

Additional differences were suggested in the 800–1100 ms epoch with an interaction between Sequence Type and Hemisphere. Follow up ANOVAs suggested main effects of Sequence Type in the left anterior and right posterior regions (all *F*s > 3.9, all *p*s < 0.05, all *η*^2^_*p*_ < 0.14). Pairwise comparisons in the left anterior region suggested that noise panels evoked a frontal positivity that was close but did not exceed the threshold of significance compared to action stars (*p* = 0.050) and explicit panels (*p* = 0.058). In the right posterior region, post-hoc comparisons implicated a greater posterior positivity to action stars than noise panels (*p* < 0.05), which is more visible in the topographic maps of Fig. 3 than in the waveforms. No differences were found between action stars and explicit panels.

### Critical panel + 1

At the panel following the manipulated, critical panel, no differences occurred between sequence types in the 200–300 ms epoch, but interactions between Sequence Type and Hemisphere appeared in the 300–500, 500–800, and 800–1100 ms epochs (Table 1), and an interaction between Sequence Type and AP Distribution appeared in the 500–800 ms epoch. Follow up analyses revealed no significant effects in regions in the 300–500 ms epoch, but main effects of Sequence Type appeared in the right posterior region for both the 500–800 and 800–1100 ms epochs (all *F*s > 6.9, all *p*s < 0.005, all *η*^2^_*p*_ < 0.23) and in the left posterior region of the 800–1100 ms epoch, *F*(2,46) = 3.6, *p* < 0.05, *η*^2^_*p*_ = 0.138. These differences arose because action stars and noise panels evoked a larger rightward posterior negativity than explicit panels (all *p*s < 0.05), which extended into the left posterior for only noise panels compared to explicit panels (*p* < 0.05).

### Individual differences

The analysis of expertise correlated only marginally with behavioral results. A positive correlation between ratings of explicit sequences and VLFI scores suggested that greater comic reading frequency lead to higher ratings, *r*(22) = 0.47, *p* < 0.05, but when correcting for multiple comparisons this correlation was no longer significant. No significant correlations were found for sequences with action stars or noise panels, nor the differences between them. No significant correlations were observed between VLFI scores and ERP results.

## Discussion

Inference has been a hallmark of the study of visual narrative processing. Here, we asked about such processing both for representationally impoverished action stars and noise panels and at the subsequent image. Our first apparent finding was that panels omitting information in a sequence elicit a large N400 effect compared to explicit depictions of an event. These N400s were evoked both by the highly conventionalized action stars and the not-conventionalized noise panels. That these inexplicit panels did not differ in amplitude of the N400 implies that they both trigger a greater demand for accessing semantic memory given the absence of overt representations. Under one interpretation, this N400 could indicate inferential processing, accessing the semantic features of the unseen information (Kuperberg et al. 2011; St. George et al. 1997). However, a simpler interpretation is that this response reflects the bottom-up activation of semantic memory (Baggio 2018; Kuperberg 2016; Kutas and Federmeier 2011) relative to the content in the prior panels, potentially in reference to expectancies of subsequent actions (Coderre et al. 2020; Magliano et al. 1996). As the bottom-up semantic content of the action stars and noise panels overlap minimally with prior panels, they trigger large N400 effects compared to explicit depictions of events.

Note that if such panels were viewed as purely asemantic, we would expect them to generate attenuated N400s compared to depictions of explicit events. This would be comparable to the findings of the lack of an N400 for action stars containing non-word symbols ($#*!) when compared to onomatopoetic or event-descriptive words in lieu of explicit events in visual narratives (Manfredi et al. 2017). Our observed effects also differ from those to blank frames that disrupted the narrative constituent structure of visual sequences, and in turn elicited left anterior negativities more associated with grammatical processing (Cohn et al. 2014). The evocation of large N400s here by action stars and noise panels suggests that meaning is being triggered, though it incurs greater cost than explicitly depicted events. However, these responses in the context of this experimental design cannot confirm whether such N400s are indicative of an inference per se.

Given that the explicit events, action stars, and noise panels differed in their representations, it was possible the N400 could have been affected by stimulus-driven physical differences between these panels. Though we attempted to control for physical differences by balancing grey values across critical panels, significant differences were found in the earlier 0–100 ms and 200–300 ms epoches. We cannot rule out the possibility that early differences affected later components. However, these earlier patterns of effects (0–100: explicit > noise/action star; 200–300: noise > explicit > action star) appear different than those observed at later epochs. In addition, despite differences prior to 300 ms, action stars and noise panels had similar amplitudes and latencies of the N400 on visual inspection, including topologically in its phasic ascent and descent. If such stimulus differences sponsored downstream effects, then these relative differences do not seem consistent across time windows in a sustained way (cf. Rossion and Caharel 2011; Taylor et al. 1999).

Despite similar N400 effects, the action star panels and noise panels differed in their subsequent processing. A late posterior positivity appeared for action stars in the later epoch, consistent with the P600 (Brouwer et al. 2016; Leckey and Federmeier 2019). Posterior positivities have been implicated in the updating of a situation model in visual narratives, for both congruous and incongruous situational changes between panels and events (Cohn 2020; Sitnikova et al. 2008). As these positivities occurred only to action stars—just like the concurrent sustained negativity—it potentially reflects the updating and/or reanalysis of the situation model of the sequence built by the context, given the incoming information of the action star (Baggio 2018; Brouwer et al. 2016; Kuperberg 2016; Van Petten and Luka 2012). Indeed, larger P600s have been observed to congruous panels with more explicit situational information (Cohn and Kutas 2015), prior to recognition of the need for an inference. However, if such a positivity reflects such conceptual integration, it would remain fairly unspecific for action stars (“an event happens”) and would still warrant further inferencing to resolve.

An alternative interpretation is that this positivity evoked only by action stars reflects a process of reanalyzing this panel’s role as a Peak in the narrative structure, whether or not integrating into a situation model (Cohn 2020; Cohn et al. 2014). This would align with P600s appearing to syntactic structure in language, and proposals that the P600 reflects processing at the interface of syntax and semantics (Brouwer et al. 2016; Michalon and Baggio 2019). Such a structural interpretation may be supported in that action stars and noise panels also differed in a left anterior distribution in the 500–800 ms epoch. This distribution is consistent with the left anterior negativity evoked by violations of narrative grammar (Cohn 2020; Cohn et al. 2014, 2012), which also have appeared along with P600s (Cohn et al. 2014; Cohn and Kutas 2017), and with similar ERPs as evoked by violations of syntactic structure in language (Bornkessel-Schlesewsky and Schlesewsky 2019; Neville et al. 1991). Both interpretations (situation model updating, narrative structure) are possibly supported by this posterior positivity only differing from noise panels, but not explicit panels. Differentiating these interpretations would require further experimentation, such as with action stars placed in other positions in a sequence.

A “lexicalized” interpretation for action stars is further implicated by their contrast with the late effects evoked by noise panels. Between 800 and 1100 ms, noise panels evoked a late frontal positivity (LFP) that was greater than both action stars and explicit panels. In research on language, LFPs have been associated with violated lexical or semantic predictions, such as when a particular word is expected in the sentence, but participants are presented with a different, yet congruent word (Leckey and Federmeier 2019; Van Petten and Luka 2012). In visual narratives, frontal positivities have been elicited in both congruous and incongruous contexts, yet with unexpected or improbable frequency (Cohn and Foulsham 2020; Cohn and Kutas 2017; Cohn and Maher 2015). Substitutions of words into image sequences also yield LFPs. When action stars contain words that index unseen events, descriptive “sound effects” (*Punch!*) elicited larger LFPs than onomatopoetic sound effects (*Pow!*), regardless of congruity (Manfredi et al. 2017). Such descriptive sound effects as a class appear less frequently than onomatopoeia in comics (Pratha et al. 2016), making them categorically less probable. That is, within the context of visual narratives, LFPs appear not only to congruous-but-unexpected stimuli, but to stimuli of any type of congruency with low probability of occurrence.

This LFP could thus be interpreted as noise panels being unexpected in the context of a visual sequence. By comparison, the lack of an LFP to action stars may affirm their conventionality as substitutive panels within a visual narrative sequence (Cohn 2019). Such findings are similar to the faster self-paced viewing times shown to action stars than empty panels, despite having more visual complexity (Cohn and Wittenberg 2015). To the extent that similar LFPs appear across sentences, visual sequences, and multimodal interactions between text and image, it could imply a domain-general response sensitive to probability of incoming forms in a given context (Leckey and Federmeier 2019; Van Petten and Luka 2012). This would align with proposals that LFPs are tied to general mechanisms like the P300, which is sensitive to attentional and probabilistic processing (Donchin and Coles 1988; Leckey and Federmeier 2019; Polich 2007). It is thus possible that, consistent with the P3a (Polich 2007), the LFP here reflects an attentional response to the unlikely, and unfamiliar, noise panels, which contrast from the conventionally recognized action stars.

It is also worth noting how these ERPs relate to previous behavioral findings (Cohn and Wittenberg 2015). In prior work, action stars had an average viewing time of ~ 650 ms, and blank panels (comparable in function to noise panels) had an average viewing time of ~ 750 ms. This means participants’ self-paced responses to advance to a subsequent panel would have just followed the processing of the N400 at these panels. Later effects (P600, LFP) would have therefore spilled over to the subsequent panel in self-paced viewing, perhaps contributing to the slower viewing times at that next position, even for action stars in sequences with scrambled panels. Nevertheless, spillover effects may not fully account for the increase in viewing times here, as we did indeed find ERP effects at the panels following action stars and noise panels.

At the panel after the critical manipulation, in the 500–800 and 800–1100 ms epochs we observed a sustained negativity with a rightward posterior distribution that was larger to panels after action stars and noise panels than event-explicit panels. While this finding implies additional differential processing between explicit and non-explicit panels, the nature of such processing is not clear by the distribution and polarity of the effect. The timing and posterior distribution is consistent with the P600 (Brouwer et al. 2016; Leckey and Federmeier 2019; Van Petten and Luka 2012), yet a P600 to panels after explicit events would be the opposite of what would be predicted. Alternatively, later negativities would be consistent with proposals of a stage of interpretive semantic processing (Baggio 2018). Sustained negativities with a frontal distribution in sentence and discourse processing have been thought to index working memory processes such as those operating to build inferred event information (Baggio et al. 2008; Bott 2010; Paczynski et al. 2014; Wittenberg et al. 2014), or search processes linking anaphors to referential information (Hoeks and Brouwer 2014; van Berkum 2009). Sustained negativities have appeared to panels in visual narratives following an inference (Cohn and Kutas 2015), like at the action star, but in an earlier epoch and a more anterior distribution. Here, we observed negativities with a slightly later latency and more posterior distribution to panels that were posited to create inferences.

An interpretation of these negativities indexing sustained working memory processes would make sense for the inference needed following a panel with impoverished event information, like action stars or noise panels. This would be consistent with proposals that sustained negativities index processes of holding information in memory to resolve ambiguities for a mental model in discourse (Baggio 2018; van Berkum 2009). This ERP pattern is consistent with behavioral findings of slower self-paced viewing times for panels following an inference, including action stars, compared to those after explicit events (Cohn and Wittenberg 2015; Huff et al. 2020; Hutson et al. 2018; Magliano et al. 2015). One such study introduced working memory load tasks between images prior to participants reaching the inference-generating panel (Magliano et al. 2015), which interfered with bridging inference generation. Interestingly, both verbal and visual working memory load tasks affected the processing of the *visual* narrative, implying a domain-general process. Nevertheless, if this sustained negativity indexes such a process, it is unclear why it has a posterior distribution here, rather than the more frontal distribution observed in prior work in both verbal and visual domains, and at the prior action star panel. Such disparity requires further work for clarity.

Finally, the lack of a difference between these negativities between panels following action stars and noise panels suggests both panels triggered similar processes. This is consistent with findings that self-paced viewing times did not differ for panels after action stars compared to blank panels (Cohn and Wittenberg 2015), which could imply similar attempts to reconcile the absence of meaningful information across these inferential techniques. In line with this, we found no difference between participants’ ratings of how much these sequences “made sense,” despite several participants recognizing the difference between action stars and noise panels in unprompted responses to post-hoc questionnaires. Thus, these panels only seemed to differ in the later effects to the panels themselves (action stars: sustained negativity/P600, noise panels: LFP), perhaps suggesting only that they departed only in recognition of their conventionality. Overall, these findings suggest that the absence of key information in a sequence may result in similar processing and assessment of their coherence, despite differences between inferential techniques themselves.

## Conclusions

This study examined the ERPs for panels which substitute for explicit events in visual narratives, thereby sponsoring bridging inferences. We observed cascading processing mechanisms similar to those observed in studies of language processing, which varied depending on the conventional (action stars) or less conventional (noise panels) properties of these panels. However, the absence of depicted events in both seemed to sponsor similar attempts to reconcile this missing information. Altogether, these findings provide an initial look at the neurocognition of inference generation across sequential images, further demonstrating that inference must balance the implicit of what is omitted and the explicit of what is provided, even when what is shown remains inexplicit.

## Data Availability

The dataset used and/or analysed during the current study are available from the corresponding author on reasonable request.
